# Influence of the Welding Process on the Mechanical Characteristics and Fracture of the S700MC High Strength Steel under Various Types of Loading

**DOI:** 10.3390/ma13225249

**Published:** 2020-11-20

**Authors:** Tadeusz Szymczak, Katarzyna Makowska, Zbigniew L. Kowalewski

**Affiliations:** 1 Department of Vehicle Type-Approval & Testing, Motor Transport Institute, 03-301 Warsaw, Poland; 2 Centre for Material Testing, Motor Transport Institute, 03-301 Warsaw, Poland; katarzyna.makowska@its.waw.pl; 3 Department of Experimental Mechanics, Institute of Fundamental Technological Research PAN, 02-106 Warsaw, Poland; zkowalew@ippt.pan.pl

**Keywords:** high strength steel, weld, HAZ, tensile curve, impact, fracture toughness, CTOD, fractography, S700MC

## Abstract

This paper focuses on the mechanical properties analysis of the high strength S700MC steel applied in welding joints. The research comprised mechanical tests for checking what the changes of tensile characteristics, mechanical parameters, resistance to impact, and fracture toughness look like in selected regions of the welding joint. Stress-strain curves have shown significant differences in the tensile characteristic shape and the values of Young’s modulus, yield stress, ultimate tensile strength, and ductility due to the welding process applied. In the case of Charpy tests, the courses of the accumulated energy, force, deflection, and project velocity are presented, indicating the maximum value of absorbed energy, the same level of force during the first contact of the projectile with the specimens, and the significant variation of the velocity for the impact energy ranging from 50 J up to 300 J. On the basis of the fracture toughness tests, the distributions of the CTOD (Crack Tip Opening Displacement) values are presented for the parent material, HAZ (Heat Affected Zone) and weld. Moreover, the characteristic features of the fatigue pre-crack, transient and crack propagation zones are identified and discussed.

## 1. Introduction

High strength steels belong to the modern engineering materials with attractive mechanical parameters from an engineering point of view. Their properties are related to the process of solid solution hardening. The treatment process is designed to take carbon out at the baking stage, creating more formable steel during plastic work and strengthening it for the applications required [1]. This stage creates a single or multi-phase microstructure represented by a ferritic single-phase or other phases such as martensite, bainite or austenite. These different microstructures represent two variants of materials, i.e., high strength steel (HSS) and advanced high strength steel (AHSS) [2]. The steels are also known as the cold-formable and commonly are denoted by adding the abbreviation: MC (M—machined hot-rolled, C—cold-formed) [3]. The multi-phase microstructure enables the unique mechanical properties and beneficial behaviour of the steel under loading to be obtained as an effect of transformation hardening [4]. It can be attained by such treatment processes as ausforming or tempforming. Their application leads to the essential differences in material behaviour under impact, providing a brittle or brittle-plastic cracking, respectively [5]. Significant microstructural effects can be observed. Among them, one can indicate the sawtooth fractures as well as the dimples of different dimensions and intensity, cleavage faces and tear ridges.

The selection of AHSS is strongly dependent on the yield stress and ultimate tensile strength. In comparison to the features of typical structural steels, these mechanical parameters are significantly higher, reaching a minimum of 700 MPa and 1300 MPa, respectively, at the ductility of 25%. The main advantages of AHSS are: (a) multi-phase structures, (b) good formability at high tensile strength, (c) higher energy absorption than that for micro-alloyed or solid solution strengthened steels, and (d) available for high and low values of the ratio of yield stress to ultimate tensile strength [4].

Thanks to the beneficial parameters, the AHSS seems to be a very attractive material for the welding joints, where in order to avoid brittle fracture, a good resistance to impact is required. The weld resistance under impact is directly dependent on the material microstructure, which is strongly related to the filler metal, parent metal (as a dilution effect) and the heat input as a linear function of the material thickness. Therefore, among the main goals of this work, one can indicate checking out whether AHSS is able to meet these requirements. As indicated by the steel manufacturer [6], the resistance of the weld on impact is often better than that presented in the catalogues of the filler metal suppliers. Good impact resistance of the weld can be obtained at low heat input, which is also necessary for the other properties of the steel to be achieved [6].

Among important issues considered in this research, one can find also tests of the main zones of the welding joint, i.e., parent material, HAZ (Heat Affected Zone) and weld. It is known that HAZ located close to the weld can have a coarse-grained zone when the toughness is low [6]. Several passes of welding and low heat input enable to limit the coarse-grained area; as a consequence, the impact resistance of HAZ can be satisfactory. The minimum mechanical requirements of the zone can be obtained if a sufficient number of welding passes are applied. For a steel sheet thickness from 6 mm to 8 mm, only three or four welding passes are sufficient. Their number increases for larger values of thickness. To produce this region with the attractive mechanical parameters, micro-jet cooling is usually used during welding [7]. The applications of AHSS in welding joints are dependent on such properties of the final product like the strength, mass, and loading capacity [8]. Due to excellent mechanical properties, it is used in many branches of industry, e.g., automotive, agriculture and forestry, etc. In the case of cars production, the steel is applied to manufacture safety cages [4,9], as well as lowbed and flatbed trailers [10,11,12]. Agriculture requires such steels for producing drawbars. AHSS is also often used in longitudinal and chassis cross beams, as well as the rear underrun protection device (RUPD) of forest timber trailers [13].

It has been found that the HAZ regions in the above-mentioned components are the major reason for the fracture development. An influence of this zone on the component behaviour can be easily identified by the hardness tests [14,15,16] if the requirements for such investigations are clearly defined [6]. A comparison of the results from such tests to the manufacturer’s claims enables assessment of the HAZ zone solely with respect to the hardness measurements. This approach is sufficient from the technological point of view, however, it is insufficient to model and predict behaviour of the welded joints under cyclic loading. To do this, additional mechanical parameters are needed, such as: elastic limit, yield stress, and ultimate tensile strength.

As presented in many papers [14,17,18,19,20], the fracture toughness tests also play an important role in the quality of welding joints assessments. It has to be noticed that the number of papers regarding complex investigations of all zones of the welding joints is still insufficient. In order to supplement the current knowledge in this area, a relatively wide experimental program was proposed in order to examine AHSS steel under different loading conditions, enabling its characterisation during tension, impact and fracture toughness tests.

## 2. Experimental Procedure

The welding joints produced from the S700MC steel were characterized using standard tensile tests, impact Charpy tests, and fracture toughness tests applying Crack Tip Opening Displacement (CTOD) methodology. The results of these experiments were supplemented by microscopic observations (conventional microscopy and SEM (Scanning Electron Microscopy, Jeol, Tokyo, Japan).

The butt joints made of S700MC steel were obtained in three passes of the welding beam. The thickness of the welded sheet was 10 mm. The welding wire OKAristoRod69 (ENISO 16834-A: GMn3Ni1CrMo) with a thickness of 1.0 mm was used during the welding process, which was carried out on the Fronius TransPuls Synergic 5000 welding device (FRONIUS INTERNATIONAL GMBH, Wels, Austria) using the MAG (Metal Active Gas) welding function with M21 shielding gas (18% CO_2_ + Argon).

The butt joints were chamfered by milling to a bevel angle of 30 deg. In the places where the joints were made, the surfaces were cleaned by grinding to a width of 20 mm. The welding parameters were as follows: remelting: 145 A, 19.3 V; filling without heating: 225 A, 27.1 V.

**Figure 1:**
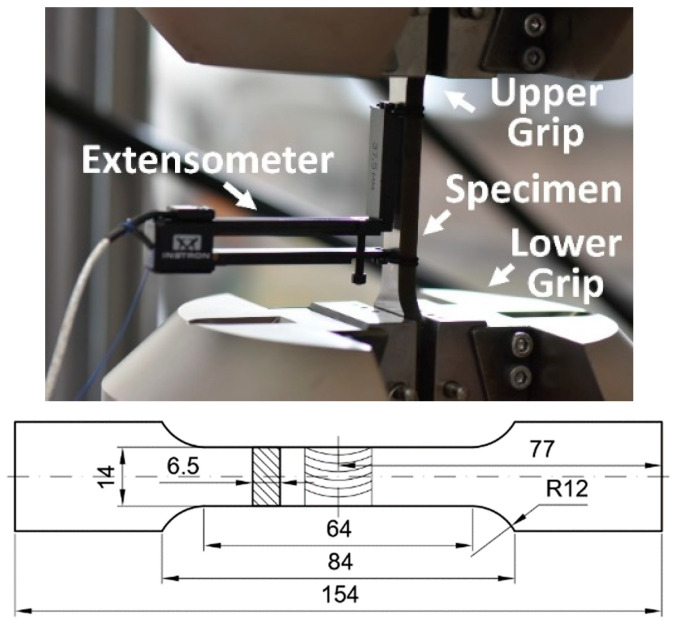
Flat specimen with weld in grips and its engineering drawing.

The monotonic tensile tests were conducted using flat specimens of total length and gauge length equal to 154 mm and 64 mm, respectively. They were manufactured from the parent material and the weld located in the middle of the specimen gauge length. In the case of parent material, a cross-section of the measurement zone was of 3.25 mm × 7 mm, while for the material in weld it was represented by the dimensions equal to 6.5 mm× 14 mm (Figure 1). A thickness of the weld was reduced by a machining process in order to adjust it to the specimen thickness defined.

The 8802 INSTRON servo-hydraulic testing machine and the 2620-601 INSTRON uni-axial extensometer (INSTRON, High Wycombe, England) of 50 mm gauge length were used in tests (Figure 1).

The Charpy experiments, characterising dynamic properties of the steel in question, were conducted at the impact energy of 50 J, 100 J, 200 J, and 300 J. In the case of the weld, the same type of tests were carried out under 100 J and 300 J.

**Figure 2:**
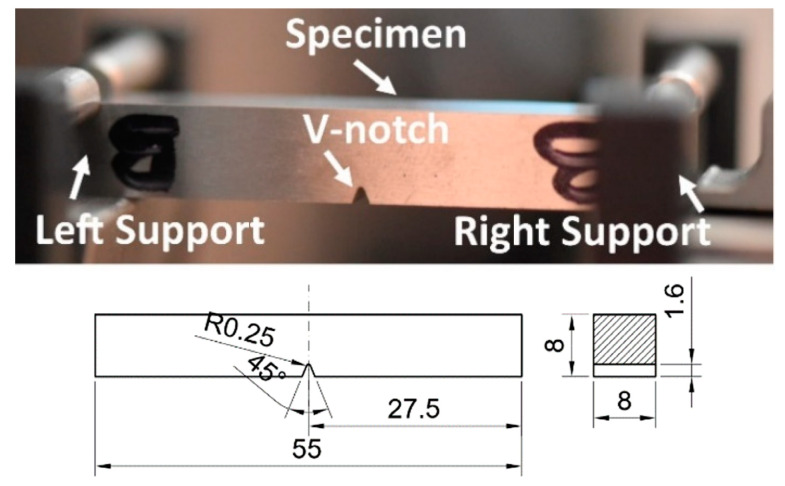
V-notched Charpy specimen in grips and its engineering drawing.

The V-notched specimens, designed according to the PN-EN ISO standards [21], were used to determine a behaviour of the material under impact (Figure 2). They had cuboidal shape of the following dimensions 8 mm × 8 mm × 55 mm, a notch of 45° angle in a tip, and 1.6 mm depth. All tests were conducted by means of the 9529 HV Instron Drop Tower at room temperature. The accumulated energy, force, projectile velocity, and deflection were collected during experiments. They were captured by means of the sensors located in the tower. Before the test, an initial value of energy must be set. It was done directly by contact of the projectile with a specimen to set zero level. Subsequently, the projectile with a mass box was automatically lifted to the position, guaranteeing the required value of the impact energy. The first value of energy was chosen experimentally under the assumption that the projectile impact should not fracture the specimen. It was equal to 50 J. Subsequent values of the impact energy up to 300 J were set by multiplication of the initial one. In a similar way, a set of impact energy levels for specimens with welds was selected.

The projectile velocity was measured by the same sensor as that used for setting the zero energy level. It was the photocell sensor located on the column of the impact tower and reference plate connected with the projectile section. Its zero value was established, taking the initial stage for the zero-energy level. The projectile velocity measurement was performed by fixing a direct contact of the projectile with a specimen and positioning the sensor to the level of the reference plate. After lifting the mass box with projectile, its drop occurred, and a time of the projectile drop was collected when the sensor beam was intersected by the reference plate. Knowing a drop distance and time of drop, a value of velocity was determined.

Each specimen after testing was subjected to microscopic observations in order to capture the characteristic features of the fracture zones.

**Figure 3:**
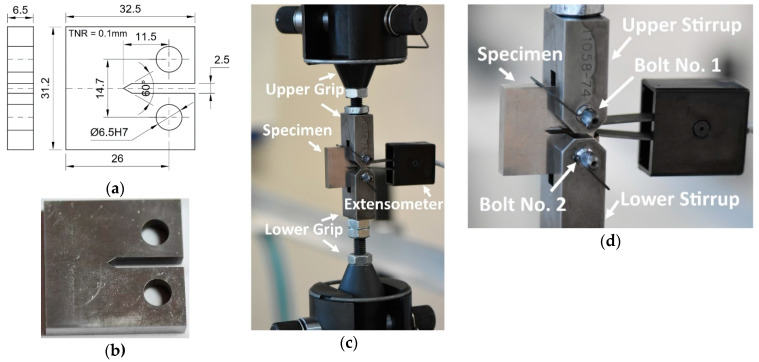
Compact tension (CT) mini-specimen: (**a**) engineering drawing; (**b**) after manufacturing; (**c**,**d**) fixed in the testing machine before experiment.

The fracture toughness experiments for CTOD determination [22,23] were designed to reflect the behaviour of the parent material, HAZ, and weld. The CT (Compact Tension) specimen geometry (Figure 3a) was elaborated based on requirements of the ASTM and PN-EN ISO Standards [24,25]. The specimens (Figure 3a,b) were prepared for all regions of the welding joint that contains the HAZ, weld as well as the parent material. They were selected from the welded sheet of the S700MC, having a thickness of 10 mm. The specimens were mounted in the special gripping system (Figure 3c,d), which guaranteed elimination of the bending moment. The Crack Opening Displacement (COD) was measured by means of the 2670-114 CTOD INSTRON extensometer (INSTRON, High Wycombe, England) of 5 mm gauge length and 2 mm displacement capacity (Figure 3d). The fatigue pre-cracking tests were conducted at the stress ratio (R = σ_min_/σ_max_, where σ_min_ and σ_max_ denote respectively, minimum stress and maximum stress during cyclic loading) equal to 0.1, up to a nominal crack length of 1.85 mm.

All specimens with weld were subjected to the machining process to remove the material surpluses in the area of the weld face and ridge, hence the measurement zones of specimens for monotonic tensile, impact tests and CTOD experiments were smooth.

The last stage of the program contains macro- and microstructural identification of the characteristic features of the regions subjected to tension, fatigue and impact.

## 3. Results and Their Discussion

**Figure 4:**
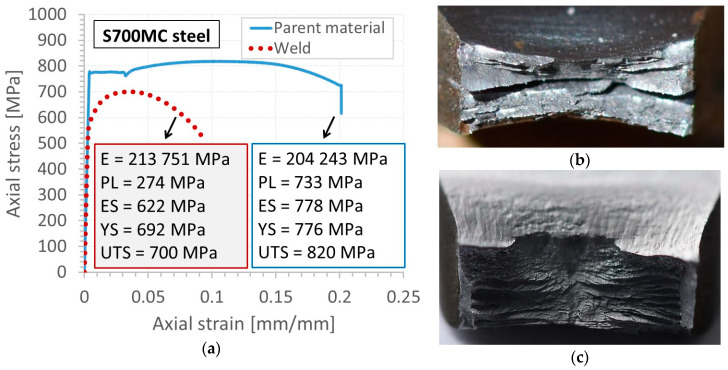
Tensile characteristics of the S700MC steel and its weld (**a**), with the fracture regions of the parent material (**b**), and the zone close to the weld (**c**).

Although the tensile curve of the S700MC steel in the as-received state exhibited elastic-plastic features with the characteristic plateau and clear hardening, it was changed significantly by the welding process. All typical mechanical parameters of this steel were reduced (Figure 4a). The greatest lowering of the mechanical properties was observed for the proportional limit, i.e., 37.5%, while the yield stress in the form of plateau disappeared entirely. The welding process also affected the fracture zones (Figure 4b,c). Contrary to the parent material fracture results that exhibited longitudinal cracks induced as an effect of the hot-rolling process of the S700MC steel (Figure 4b), the fracture region close to the weld was more uniform (Figure 4c). It indicates that the typical crack features disappeared due to temperature exposure during the joining technology applied (Figure 4). Nevertheless, low-depth cleavage facets were observed. They were relatively densely distributed.

**Figure 5:**
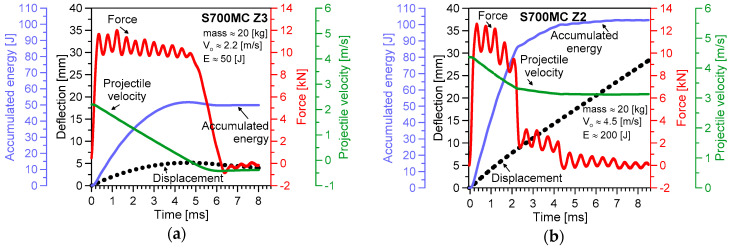
Variations of the Charpy test parameters for the S700MC steel (parent material) under impact energy of: (**a**) 50 J; and (**b**) 200 J.

**Figure 6:**
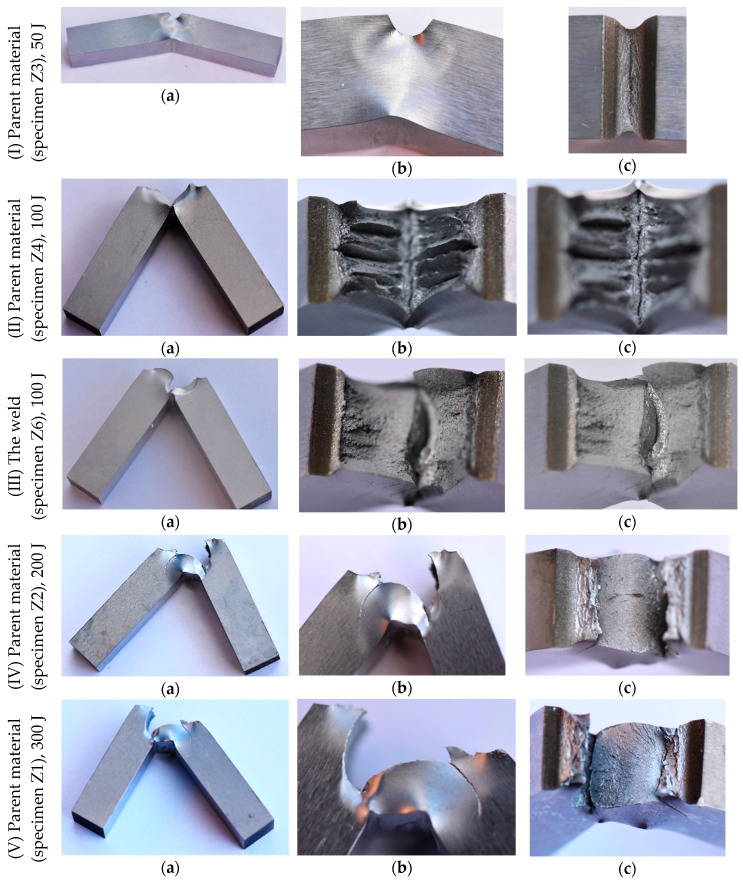

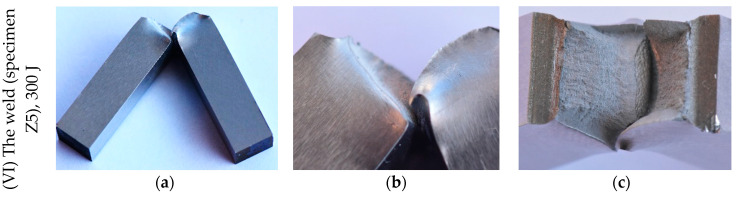
V-notched specimens of the parent material and weld of S700MC steel after Charpy test: (**a**) —specimen after test; (**b**,**c**)—fracture zone from side and top view, respectively.

**Figure 7:**
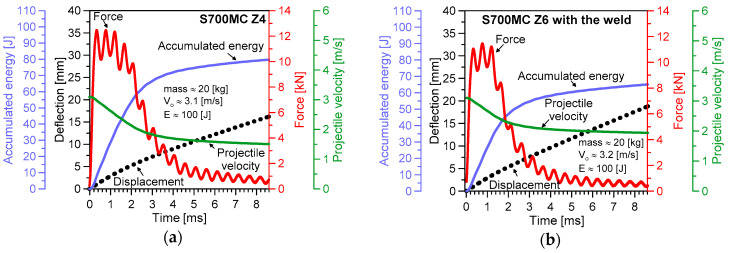
Variations of the Charpy test parameters for the S700MC steel: (**a**) parent material; (**b**) weld; (impact energy: 100 J).

**Figure 8:**
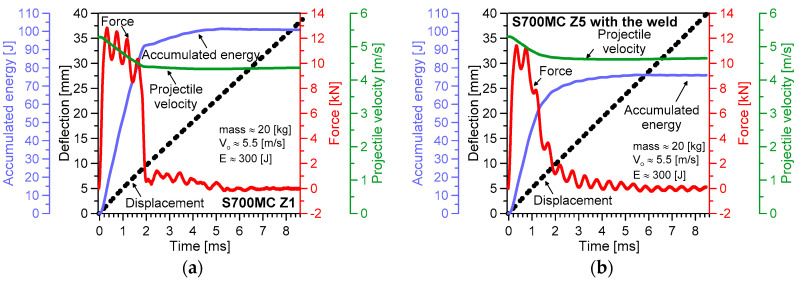
Variations of the Charpy test parameters for the S700MC steel: (**a**) parent material; (**b**) weld (impact energy: 300 J).

**Figure 9:**
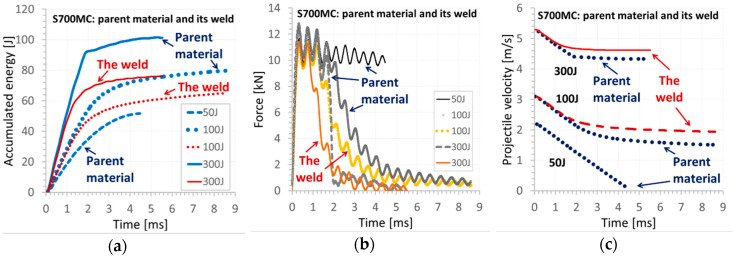
Variations of the following parameters: (**a**) accumulated energy; (**b**) force; (**c**) projectile penetration velocity for the parent material (S700MC steel) and its weld during impact tests.

The results of the impact tests are presented in Figure 5, Figure 6, Figure 7, Figure 8 and Figure 9. The maximum accumulated energy for the parent material reached 100 J, defining the limit value of the absorbed energy for the range of the impact energy applied (Figure 9a). The variations of deflection were represented by a quasi-linear relationship independently of the energy applied in the tests, and thus, reflected a stable response of the steel tested under the impact (Figure 5, Figure 6, Figure 7 and Figure 8). Force variations one can interpret as the damping reduction with the energy-applied increase (Figure 9b). In the case of the projectile velocity, its linear course was disturbed for the impact energy higher than 50 J (Figure 9c).

It has to be noticed that specimens were not completely broken for all values of impact energy applied, indicating the significant resistance of the steel tested into the loading types used (Figure 6). Some differences in the material behaviour can be evidenced by the analysis of the fracture regions. The longitudinal cracks were clearly visible in the case of the parent material subjected to an impact of 100 J (Figure 6(II)b,c). They were perpendicular to the direction of the impact force and similar to the other ones obtained during the tensile test (Figure 4). Such effect was not observed at higher values of the energy applied, e.g., 200 J and 300 J (Figure 6(IV)b,c, and Figure 6(V)b,c, respectively). It is easy to notice on the force–time characteristics, that at the lower energy a gradual reduction of the force occurred, while in the case of the larger values of energy, a rapid drop of the force appeared (Figure 5b and Figure 8a). It is presumably connected with the mechanism of degradation change from the brittle to plastic, and additionally, reflected by a clear variation of the course of accumulated energy from the linear to non-linear.

The fracture zone of the weld expressed different character than that for the parent material obtained. A smooth surface was observed at 100 J (Figure 6(III)b,c) and 300 J (Figure 6(VI)b,c). For such energy levels, the material fracture was strongly dependent on the velocity. A comparison of the results achieved confirmed that the parent material exhibited better resistance than that for the welding joint obtained (Figure 9). This was clearly reflected by the higher values of accumulated energy, longer time of the force action, and larger values of the projectile velocity for the weld material in comparison to the parent one (Figure 9).

**Figure 10:**
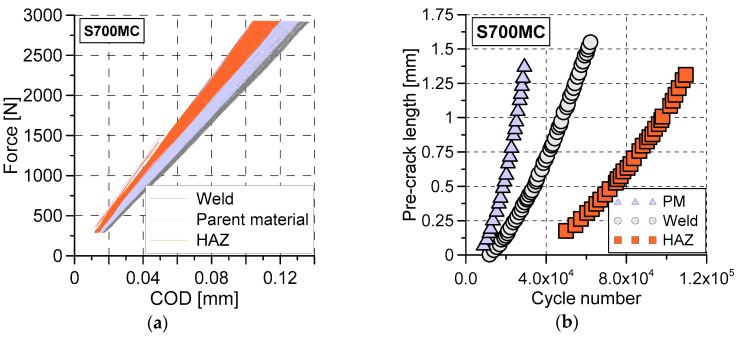
Results of CTOD experiments: (**a**) force versus COD; (**b**) pre-crack length-cycle number for the parent material (PM), weld and HAZ.

**Figure 11:**
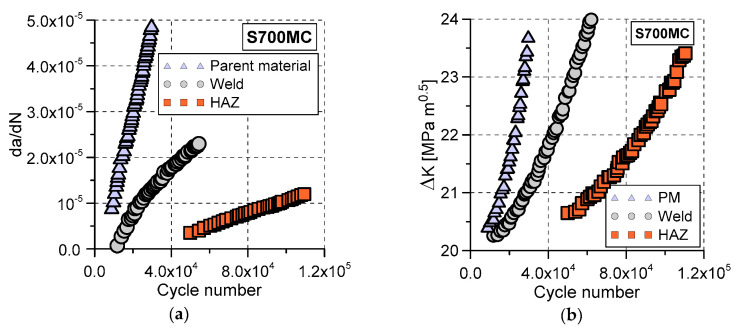
Variations of the crack propagation parameters: as a function of the cycle number for the parent material, weld and HAZ: (**a**) da/dN; (**b**) ∆K.

**Figure 12:**
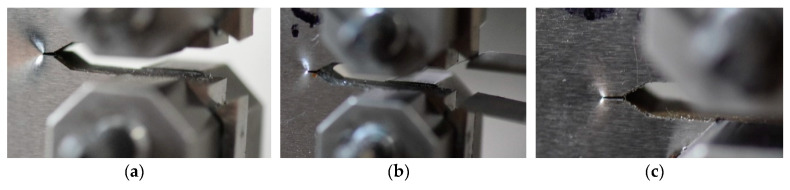
CT mini-specimens before the propagation of the fatigue pre-crack: (**a**) parent material; (**b**) HAZ; (**c**) weld.

**Figure 13:**
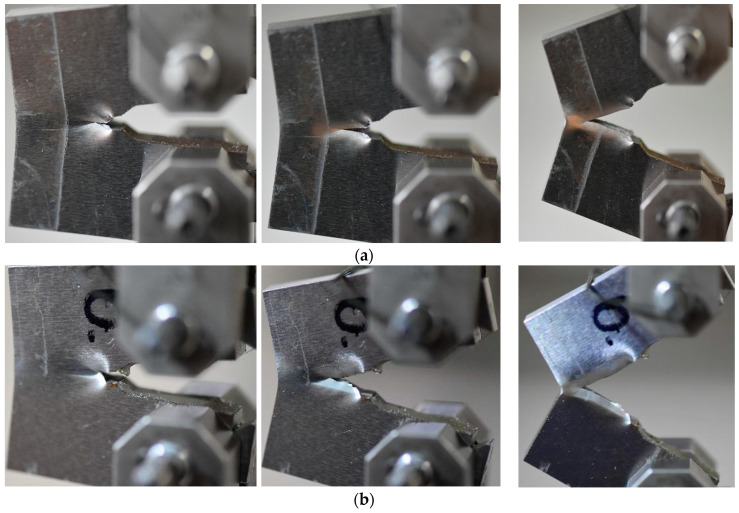

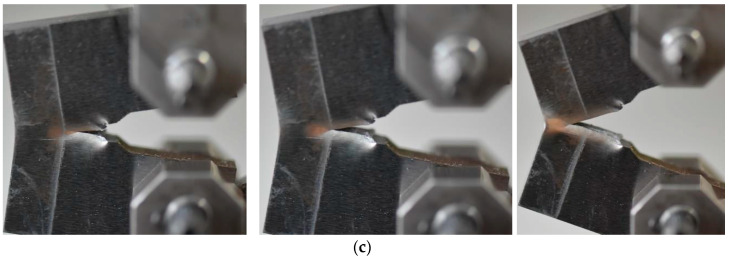
CT mini-specimens at the final stage of CTOD tests: (**a**) parent material; (**b**) HAZ; (**c**) weld.

**Figure 14:**
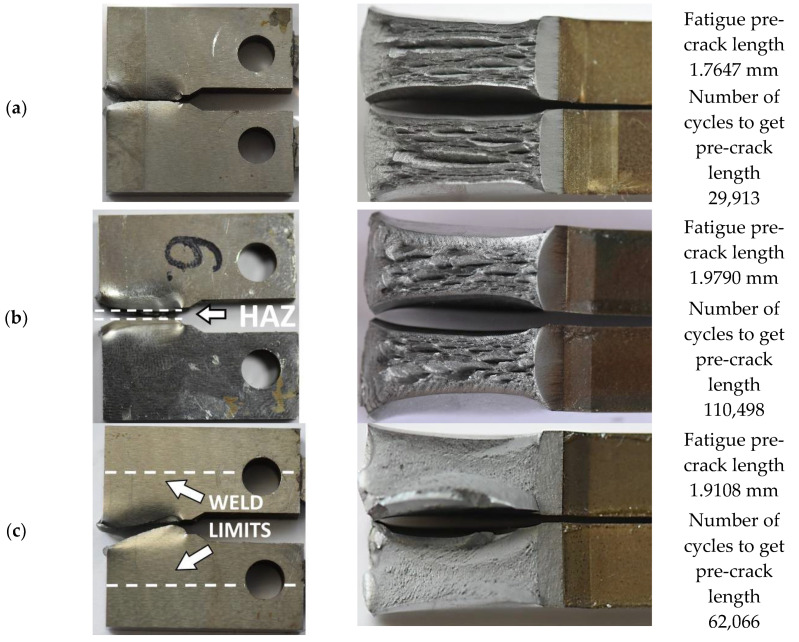
CT mini-specimens after CTOD tests: (**a**) parent material; (**b**) HAZ; (**c**) weld.

The influence of the welding on the steel behaviour can also be demonstrated on the basis of the fracture test results: force versus COD during the pre-cracking stage (Figure 10a), crack length (Figure 10b), its propagation rate (Figure 11a), and ∆K (stress-intensity factor range, ∆K = Kmax − Kmin) [26] (Figure 11b) versus the number of cycles as well as photos of the CTOD specimens at various stages of tension (Figure 12 and Figure 13). The following data were considered: fatigue pre-crack, transient and crack propagation zones of the regions investigated: parent material, HAZ and weld (Figure 14), and variations of force versus COD.

The highest stiffness for the HAZ specimen and the lowest one for the weld (Figure 10a) were identified during the pre-cracking fatigue stage. The number of cycles necessary to create the required length of the fatigue pre-crack and its propagation rate reached the greatest value (Figure 10a and Figure 11a) for the HAZ region. Taking into account the welding quality, this is a beneficial effect. It means that the probability of cracks occurrence in this region is much lower than that in other zones tested.

A similar character of the results can be observed in analysis of ∆K variations (Figure 11b). A comparison of the fatigue data for three tested zones indicated that the HAZ region exhibited the highest cyclic loading resistance. Before the fatigue cracks development, three regions in question demonstrated similar ability to crack propagation along two different directions (Figure 12), however, during tension, the major crack propagated only in a single direction, which was orientated horizontally (Figure 13).

Significant differences in features of the zones taken into account can be indicated on the crack propagation surfaces (Figure 14). They are represented by a gradual vanishing of the longitudinal cracks depending on the zone. In the case of the parent material, the number of cracks was the largest, while for the weld it was almost imperceptible. Although the HAZ zone exhibited almost similar features to the parent material, the crack lengths of both zones were different, significantly shorter in the HAZ than those in the parent material observed.

**Figure 15:**
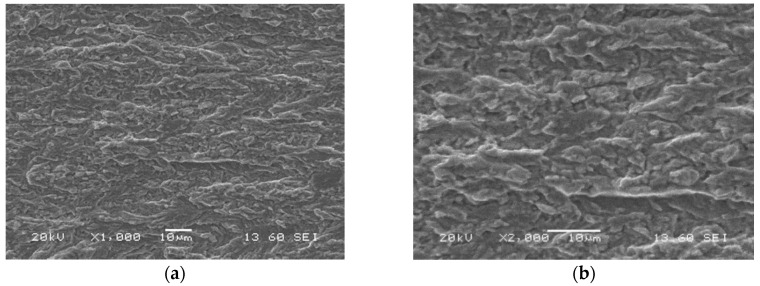
Microstructures of the fatigue pre-crack zone of the parent material (specimen No. 7) captured after CTOD test using SEM; magnifications: (**a**) ×1000; (**b**) ×2000.

Figure 15 presents the SEM images of the fatigue pre-crack zone of the S700MC with visible segmental fatigue fringes randomly distributed. The features of the fatigue regions (Figure 15) were different than those for other structural steels, namely, the fatigue fringes were distributed irregularly.

**Figure 16:**
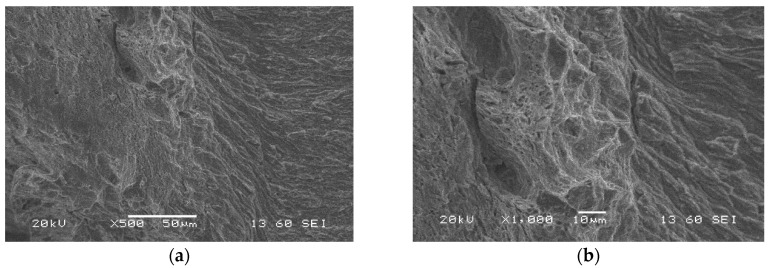
Microstructures of the transient region (fatigue pre-crack and crack propagation) of the S700MC (specimen No. 7) captured after CTOD test using SEM; magnifications: (**a**) ×500; (**b**) ×1000.

Figure 16 shows the transition region (fatigue pre-crack and crack propagation) between two types of fracture. Also, the crack propagation direction is well visible. The lamellar fracture with very fine particle morphology was obtained. The lamellar character of the fracture corresponds to the anisotropic microstructure of the base material. It proves the entire removal of dislocation reinforcement after thermomechanical treatment [27]. The transient region expressed features of typical solid bodies that differ themselves in cracking type from brittle to brittle-plastic (Figure 16). It seems, that in the case of steel tested, the fatigue crack that grows under tension is similar to that of typical steels.

The transition zone reflected the material fracture in the stage preceding decohesion (Figure 16). It had the features representing coupled (brittle-plastic) cracking located in the entire area considered (Figure 16). One can conclude that the fatigue crack growth under tensile conditions is controlled by the same material degradation mechanisms as those for typical steel grades observed. Additionally, a lack of the main plane of damage development can be easily indicated.

**Figure 17:**
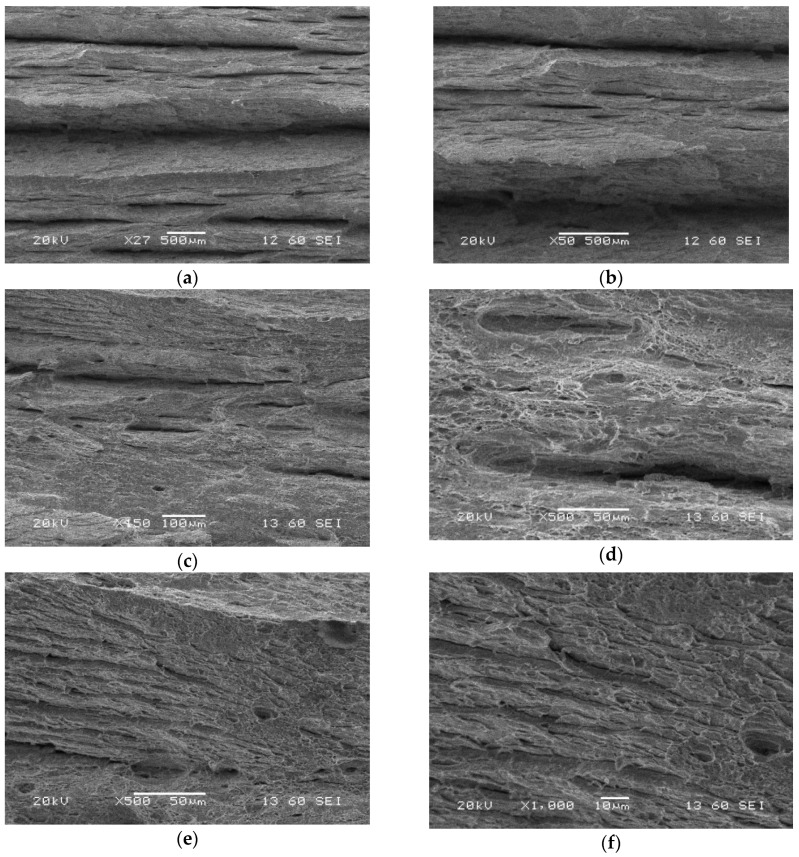
Microstructures of the crack propagation zone of the parent material (specimen No. 7) captured after CTOD test using SEM; magnifications: (**a**) ×27; (**b**) ×50; (**c**) ×450; (**d**) and (**e**) ×500; (**f**) ×1000.

The crack propagation zone of the parent material was captured using SEM in order to determine its significant features (Figure 17). Cleavage facets between defects looking like delamination were identified. Delamination-like fracture of the steel was the main feature of the zone examined (Figure 14a and Figure 17a,b). This effect exhibited the same orientation in the area inspected. The same effect can be indicated for the HAZ, however, the number of longitudinal cracks was smaller (Figure 14b). It disappeared in the weld as a result of the joining process (Figure 14c).

**Figure 18:**
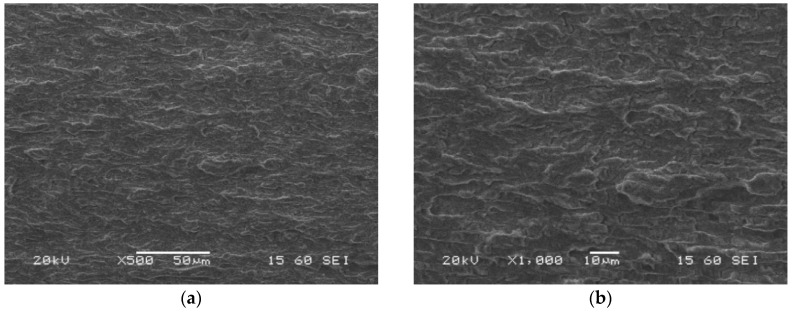
Microstructures of the fatigue pre-crack zone of the HAZ (specimen No. 6) captured after CTOD test using SEM; magnifications: (**a**) ×500; (**b**) ×1000.

**Figure 19:**
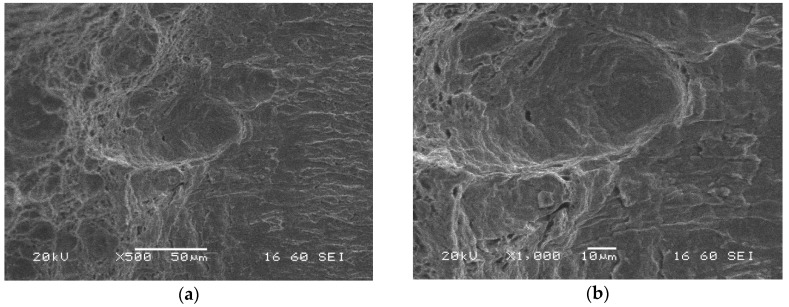
Microstructures of the transient (fatigue pre-crack and crack propagation) region of the HAZ (specimen No. 6) captured after CTOD test using SEM; magnifications: (**a**) ×500; (**b**) ×1000.

**Figure 20:**
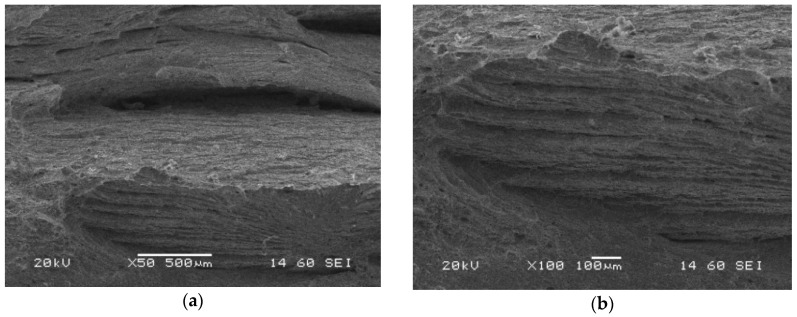
Microstructures of the crack propagation zone of the HAZ (specimen No. 6) captured after CTOD test using SEM; magnifications: (**a**) ×50; (**b**) ×1000.

Similar features of the fatigue pre-crack zone (Figure 18), transition region (Figure 19) and crack propagation (Figure 20) were observed either for the parent material or HAZ. However, the characteristic effects noticed previously for each part of the fracture are visible at a lower scale. Secondary cracks and striations in the fatigue pre-crack zone (Figure 18) as well as the delamination-like areas in the crack propagation zone (Figure 20) exhibit smaller dimensions. In the area of the transient region of the HAZ, larger perpendicular cracks appear in the loading direction (Figure 19). These changes in the fracture morphology can be explained by the carbide re-precipitation and microstructure homogenisation as a result of heat input accompanying the welding process.

**Figure 21:**
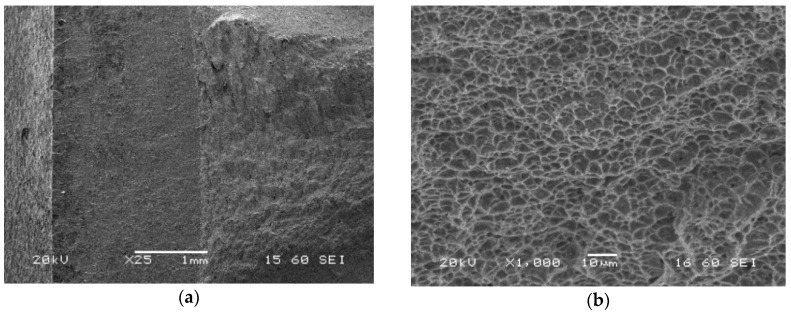
Microstructures of the zone located in the transition region of the weld (specimen No. 3) obtained during CTOD test using SEM; magnifications: (**a**) ×25; (**b**) ×1000.

Contrary to these results, the zone was represented by the same features in the case of the weld, however, at a lower scale and with more regular distribution (Figure 21). The transient zone of the weld presents classical dimples that prove the ductile character of the region considered. The fracture morphology is isotropic without a delamination-like effect.

**Figure 22:**
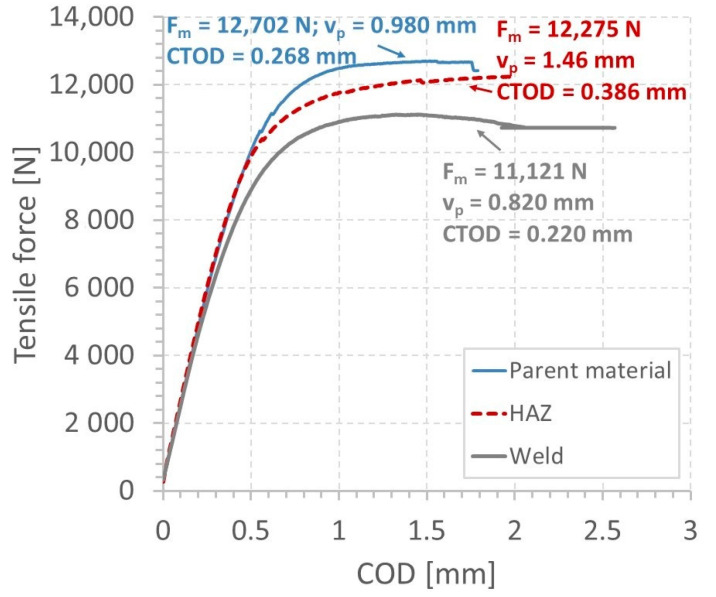
Force versus COD for: the parent material, HAZ and weld.

Despite the unstable crack growth in HAZ, the CTOD parameter in this zone took the highest value. It was equal to 0.386 mm (Figure 22). In comparison to values calculated for the parent material and weld, it was 37% higher.

Taking into account the results of the entire experimental program carried out, one can notice a great usefulness of each testing technique used in this research. The data achieved by means of these techniques in many aspects are complementary to each other, thus giving more thorough knowledge on the welding joints’ quality. It can be particularly suitable for engineers working on the development of new technologies of welding. It was shown how important are the microscopic observations in analysis of macroscopic investigations. Thanks to them, it was easy to distinguish the representative features of all typical zones of the welding joint and assign them to the observed changes in parameters determined from macroscopic techniques. Variations of selected physical parameters characterizing the impact strength like the accumulated energy or projectile velocity during Charpy test can be attributed to the microscopic effects. For example, in the case of low impact energy associated with gradual reduction of the force, the specimen was slightly deformed without clear fracture effects. For higher impact energies, the force dropped much faster, and as a consequence, the specimen exhibited marked fracture. As it has been shown in this research, the type of the fracture was directly related to the impact energy on the one hand, and the zone tested (parent material or weld) on the other. In the case of the impact energy equal to 100 J, the fracture of the parent material specimen contained several longitudinal, quite long and deep voids looking like delaminated material. For impact energy higher than 100 J, the effect totally disappeared. For the weld material, similar behaviour was observed, however, for the impact energy equal to 100 J, the voids were much shorter and significantly shallower.

Effect looking like a delamination appeared also during CTOD tests, and again, it was directly connected with the macroscopic parameters of tests and the type of welding zone considered. One can indicate the delamination-like effect in the crack propagation zone and a lack of fringes in the fatigue pre-crack regions for the parent material and HAZ. The welding process led to significant reduction of the effect, which was clearly observed for the specimen containing the weld.

## 4. Concluding Remarks

The experimental program enabled characterization of the S700MC steel behaviour applied in the welding joint as the parent material; the material of heat affected the zone and weld on the basis of the standard tensile test, dynamic impact and fracture toughness tests. After all of these tests, microscopic analyses were performed providing important data for better understanding of the fracture process development. The results enable to formulate some important remarks and conclusions:

1. The mechanical properties of the S700MC steel were very sensitive to the welding process. As a consequence of welding, the yield stress, ultimate tensile strength and ductility were reduced significantly.
2. The maximum energy accumulated in the S700MC steel during impact reached 100 J. Independently of the impact energy applied, the maximum values of the force were very similar. The features of fracture regions after impact were strongly related to the energy level applied.
3. Physical parameters characterizing the impact strength of the material like the accumulated energy or projectile velocity were changed significantly due to the welding process applied.
4. The welding process led to a reduction of the delamination-like effect, an increase of the cleavage facets and dimples in each tested zone.

On the basis of this experimental work, some guidelines may be formulated for the future research, namely, in order to guaranty better assessments of the welding joints, the fracture toughness tests should be conducted in each characteristic zone of the joint. To improve contemporary welding technology, its development should be more closely coupled with the advanced testing in the framework of fracture mechanics and multi-scale microstructural analysis.

It has to be emphasized finally that the statistical issues played here are of secondary importance, since only a single type of welding joint was considered. It represents the welding joint of two sheets, where the welding head (that passed from the beginning of the element to its end) had the constant process parameters. Moreover, the number of specimens was limited due to the relatively small total length of the joint delivered by the company interested in this research.

## References

[1] Billur E., Altan T. (2013). Three generations of advanced high-strength steels for automotive applications. Part I, An FMA Publication. https://ercnsm.osu.edu/sites/default/files/uploads/664-3.pdf.

[2] (2017). Advanced High-Strength Steels for Automotive; Automotive Technology, Automotive Innovations Engineering & Technology. https://www.automotive-technology.com/.

[3] (2015). Strenx 700 MC Advanced High Strength Steel.

[4] Horvath C.D. (2004). The Future Revolution in Automotive High Strength Steel Usage.

[5] Dolzhenko A., Yanushkevich Z., Nikulin S., Belyakov A., Kaibyshev R. (2018). Impact toughness of an S700MC-type steel: Tempforming vs ausforming. Mater. Sci. Eng..

[6] (2015). Welding of Strenx Advanced High Strength Steels.

[7] Silva A., Szczucka-Lasota B., Węgrzyn T., Jurek A. (2020). Welding of DOCOL 1200M using micro-jet cooling. Weld. Technol. Rev..

[8] (2016). The Beauty.

[9] Hall J.N. (2011). Evolution of Advanced High Strength Steels in Automotive Applications.

[10] (2019). Van Trailers.

[11] Keeler S., Kimchi M., Mconey P.J. (2017). Advanced High-Strength Steels Application Guidelines.

[12] Fentahun M.A., Savas M.A. (2018). Materials Used in Automotive Manufacture and Material Selection Using Ashby Charts. Int. J. Mater. Eng..

[13] (2019). Timber Trailers.

[14] Szymczak T., Brodecki A., Makowska K., Kowalewski Z.L. (2019). Tow truck frame made of high strength steel under cyclic loading. Mater. Today Proc..

[15] Kurc-Lisiecka A., Piwnik J., Lisiecki A. (2017). Laser welding of new grade of advanced high strength steel Strenx 1100 MC. Arch. Met. Mater..

[16] Atta-Agyemang S.A., Kesse M.A., Kah P., Martikainen J. (2016). Improvement of strength and toughness: The effect on the weldability of high-strength steels used in offshore structures. Proc. Inst. Mech. Eng. Part B J. Eng. Manuf..

[17] Nevasmaa P., Karjalainen-Roikonen P., Laukkanen A., Nykänen T., Ameri A., Björk T., Limnell T., Kuoppala J. Fracture characteristics of new ultra-high-strength steel with yield strengths 900–960 MPa. Proceedings of the 2nd International Conference Super-High Strength Steels (SHSS), Peschiera del Garda.

[18] Ismar H., Burzic Z., Kapor N.J., Kokelj T. (2012). Experimental Investigation of High-Strength Structural Steel Welds. J. Mech. Eng..

[19] Dzioba I., Pała T., Valkonen I. (2013). Strength and fracture toughness of the welded joints made of high-strength ferritic steel. Acta Mech. Autom..

[20] Chabok A., Galinmoghaddam E., De Hosson J.T.M., Pei Y.T. (2019). Micromechanical evaluation of DP1000-GI dual-phase high-strength steel resistance spot weld. J. Mater. Sci..

[21] (2016). Metallic Materials–Charpy Pendulum Impact Test–Part 1: Test Method.

[22] (2008). Standard Test Method for Crack-Tip Opening Displacement (CTOD) Fracture Toughness Measurement.

[23] (2020). Standard Test Method for Measurement of Fracture Toughness.

[24] (2012). Standard Test Method for Linear-Elastic Plane-Strain Fracture Toughness KIc of Metallic Materials.

[25] (2011). Metallic Materials-Determination of Plane-Strain Fracture Toughness.

[26] (2015). Standard Test Method for Measurement of Fatigue Crack Growth Rates.

[27] Schmidová E., Bozkurt F., Culek B., Kumar S., Kuchariková L., Uhríčik M. (2019). Influence of welding on dynamic fracture toughness of Strenx 700MC steel. Metals.

